# Salivary Thromboxane A2-Binding Proteins from Triatomine Vectors of Chagas Disease Inhibit Platelet-Mediated Neutrophil Extracellular Traps (NETs) Formation and Arterial Thrombosis

**DOI:** 10.1371/journal.pntd.0003869

**Published:** 2015-06-25

**Authors:** Daniella M. Mizurini, Jorgeane S. Aslan, Tainá Gomes, Dongying Ma, Ivo M. B. Francischetti, Robson Q. Monteiro

**Affiliations:** 1 Instituto de Bioquímica Médica Leopoldo de Meis, Universidade Federal do Rio de Janeiro, Rio de Janeiro, Brazil; 2 Vector Molecular Biology Section, Laboratory of Malaria and Vector Research (LMVR), National Institute of Allergy and Infectious Diseases (NIAID), National Institutes of Health (NIH), Bethesda, Maryland, United States of America; United States Food and Drug Administration, UNITED STATES

## Abstract

**Background:**

The saliva of blood-feeding arthropods contains a notable diversity of molecules that target the hemostatic and immune systems of the host. Dipetalodipin and triplatin are triatomine salivary proteins that exhibit high affinity binding to prostanoids, such as TXA2, thus resulting in potent inhibitory effect on platelet aggregation *in vitro*. It was recently demonstrated that platelet-derived TXA2 mediates the formation of neutrophil extracellular traps (NETs), a newly recognized link between inflammation and thrombosis that promote thrombus growth and stability.

**Methodology/Principal Findings:**

This study evaluated the ability of dipetalodipin and triplatin to block NETs formation *in vitro*. We also investigated the *in vivo* antithrombotic activity of TXA2 binding proteins by employing two murine models of experimental thrombosis. Remarkably, we observed that both inhibitors abolished the platelet-mediated formation of NETs *in vitro*. Dipetalodipin and triplatin significantly increased carotid artery occlusion time in a FeCl_3_-induced injury model. Treatment with TXA2-binding proteins also protected mice from lethal pulmonary thromboembolism evoked by the intravenous injection of collagen and epinephrine. Effective antithrombotic doses of dipetalodipin and triplatin did not increase blood loss, which was estimated using the tail transection method.

**Conclusions/Significance:**

Salivary TXA2-binding proteins, dipetalodipin and triplatin, are capable to prevent platelet-mediated NETs formation *in vitro*. This ability may contribute to the antithrombotic effects *in vivo*. Notably, both molecules inhibit arterial thrombosis without promoting excessive bleeding. Our results provide new insight into the antihemostatic effects of TXA2-binding proteins and may have important significance in elucidating the mechanisms of saliva to avoid host’s hemostatic responses and innate immune system.

## Author Summary

Chagas disease is transmitted by the protozoan parasite *Trypanosoma cruzi*. The main form of transmission in endemic areas involves a life cycle in which blood-sucking triatomine vectors get infected by biting an infected animal or person. The saliva of blood-feeding arthropods contains a remarkable diversity of molecules that target the hemostatic and immune systems of the host. Thus, the systematic study and characterization of salivary proteins constitutes a strategy for identifying new exogenous compounds that may serve as prototypes for development of new drugs as well as strategies for vector control. Our group has studied the antihemostatic and antithrombotic properties of several exogenous inhibitors. In this report we demonstrated that the TXA2-binding proteins, dipetalodipin and triplatin, impair platelet-assisted formation of neutrophil extracellular traps (NETs). NETs have been described as web-like structures of DNA and proteins that play an important role in killing of pathogens. In addition, NETs have been recently implicated in thrombus formation. According to this, we demonstrate here that dipetalodipin and triplatin exhibit antithrombotic activity in two distinct *in vivo* mice models that are highly dependent on platelets. Remarkably, both molecules inhibited thrombosis without promoting excessive bleeding. Altogether, our results provide new insight into the antihemostatic effects of TXA2-binding proteins and may help to elucidate the mechanisms of saliva to avoid host’s hemostatic responses and innate immune system.

## Introduction

To take a blood meal, triatomine bugs pierce the host skin searching for a blood vessel, which causes tissue damage and elicits the hemostatic response of the vertebrate host against blood loss. The first mechanism of vertebrate defense to counteract blood loss is constituted by platelet aggregation that forms the primary hemostatic plug. Following vascular injury, a number of extracellular matrix proteins, such as collagen and von Willebrand factor (vWF), are exposed to flowing blood, thus initiating platelet adhesion [1]. The initial tethering induces platelet deceleration and “rolling” along the exposed extracellular matrix until stable adhesion can occur. This activation causes a cytoskeletal reorganization to change the platelet shape and cover a larger surface area at the site of damage. It also induces intracellular signaling, leading to cellular activation and the release of second wave mediators, such as adenosine diphosphate (ADP) and thromboxane A2 (TXA2), that amplify the activation signal and recruit additional platelets to the growing thrombus [2,3]. TXA2 is synthesized from membrane-released arachidonic acid during platelet activation and plays an important role in the positive feedback for activation and the recruitment of additional platelets to the primary hemostatic plug, thus contributing to thrombus formation [4].

Salivary glands from hematophagous animals constitute a major source of molecules capable of modulating hemostasis [5–7]. Blood-sucking-derived antihemostatic molecules are comprised of a notable diversity of platelet aggregation inhibitors, including enzyme inhibitors, nitric oxide (NO)-releasing molecules, integrin antagonists, apyrases, collagen-binding proteins and molecules that bind biogenic amines [6,8]. Dipetalodipin and triplatin, two salivary proteins belonging to the lipocalin family, have been recently characterized as high-affinity prostanoid-binding proteins that modulate platelet function, vasoconstriction, and angiogenesis [9,10]. Remarkably, both proteins are potent TXA2 scavengers, which explain their inhibitory effects on platelet aggregation induced by low concentrations of collagen, arachidonic acid and the TXA2 mimetic (U46619).

In addition to hemostasis, the host’s response against tissue injury involves recruitment of inflammatory cells [5]. Neutrophils constitute the first line of defense against infection, since they are involved in phagocytosis and the intracellular degradation of invading microorganisms [11] or creating an extracellular environment to kill pathogens by a mechanism involving neutrophil extracellular traps (NETs) [12]. NETs have been described as web-like structures of DNA and proteins form through a process called NETosis [13] and they have been recently linked to blood coagulation [14] and platelet activation [15]. It is proposed that platelets play a relevant role in neutrophil functions [16,17]. In this context, it has been recently described that platelet-induced NET formation depends on the production of TXA2 [18].

In this study, we investigated the *in vivo* effects of dipetalodipin and triplatin on thrombus formation using two distinct mice models. Remarkably, both molecules inhibited arterial thrombosis and collagen-induced thromboembolism at doses that caused no bleeding effects. In addition, dipetalodipin and triplatin abolished the platelet-mediated formation of NETs. We conclude that TXA2 scavenger might represent an important mechanism of action of saliva to avoid host’s hemostatic responses and innate immune system.

## Materials and Methods

### Ethics statement

Blood products used in this study were obtained from the Blood Bank at the University Hospital Clementino Fraga Filho from the Federal University of Rio de Janeiro (Rio de Janeiro, Brazil). Blood donation was obtained from healthy adult subjects upon written informed consent. The use of blood products for research was further approved upon oral informed consent due to the elevated number of specific research projects and because the risks were low and the potential harm for participants was unlikely. Oral consent for the use of plasma and blood cells in this study was approved by The Committee for Ethics in Human Research (CEP-HUCFF/FM 213/07). The oral consent was documented in an appendix form of the blood donation written consent that states: “I also, authorize that the surplus of samples and cells of the bags, when not indicated to be applied in clinical can be used in research in basic sciences for health promotion. I am aware that research projects will be selected by the technical employee responsible for the transfusion service, with the criterion of being proven by the rules approved by the research ethics in Brazil, through the authorized organism—the National Council of Ethics (CONEP).” All animal care and experimental protocols were conducted following the guidelines of the institutional care and use committee (Committee for Evaluation of Animal Use for Research from the Federal University of Rio de Janeiro, CAUAP-UFRJ) and the NIH Guide for the Care and Use of Laboratory Animals (ISBN 0-309-05377-3). The protocols were approved by CAUAP-UFRJ under registry #IBQM/081-05/16. Technicians dedicated to the animal facility at the Institute of Medical Biochemistry (UFRJ) carried out all aspects related to mouse husbandry under strict guidelines to insure careful and consistent handling of the animals.

### Chemicals

Recombinant dipetalodipin and triplatin were produced in *Escherichia coli*, purified, and quantified as described previously [9,10]. Standard collagen (equine fibrillar type I Horm [type I/H]), was obtained from the Chrono-Log Corp. (Haverstown, PA, USA). Anasedan (xylazin) and Dopalen (ketamin) were purchased from Agribrands (Rio de Janeiro, RJ, Brazil). Epinephrine, HISTOPAQUE solution (10771), phorbol myristate acetate (PMA), L-α-phosphatidylcholine (PC), and L-α-phosphatidylserine (PS) were purchased from the Sigma Chemical Co. (St. Louis, MO, USA). A rabbit polyclonal antibody against histone H3 (citrulline R2 + R8 + R17; ab5103) was from Abcam (San Francisco, CA, USA) and a goat anti-rabbit IgG labeled with Alexa 488 was from Molecular Probes (São Paulo, SP, Brazil). Hoechst 33342 was purchased from Life Technologies (São Paulo, SP, Brazil). Phospholipid vesicles (PC/PS) composed of 75% PC/25% PS (w/w) were prepared by sonication. Briefly, phospholipids in chloroform were dried with a N_2_ stream and lyophilized. The lipids were resuspended in 50 mM Tris-HCl and 150 mM NaCl (pH 7.5) sonicated for 10 min and adjusted to a final concentration of 500 μM.

### Clotting assays

The effect of dipetalodipin and triplatin on collagen-induced plasma clotting was evaluated on an Amelung KC4A coagulometer (Labcon, Heppenheim, Germany) as previously described [19] with slightly modifications. Coagulation time was monitored in human citrate-anticoagulated platelet-rich plasma (PRP) or in platelet-poor plasma (PPP) supplemented with 10 μM PC/PS. Human blood samples were collected from healthy donors in 3.2% trisodium citrate (9:1, v/v); PRP was obtained by centrifugation at 800 × g for 10 min and PPP was obtained by further centrifugation of the PRP at 2,000 × g for 10 min. Briefly, dipetalodipin (1 μM) or triplatin (2 μM) was incubated with collagen (50 μL) for 10 min, at 37°C, before adding 50 μL of PRP or PPP containing PC/PS. After 10 min, clotting was triggered by the addition of CaCl_2_ (16.6 mM, final concentration).

### Preparation of washed human platelets

Whole blood from healthy donors was obtained by venipuncture in 3.2% sodium citrate (9:1, v/v). Warmed ACD (85 mM sodium citrate, 110 mM glucose, 71 mM citric acid) was added to the blood (1:9 v/v ACD to anticoagulated whole blood) and centrifuged for 10 min, at 200 × g, at room temperature. The supernatant platelet-poor plasma (PPP) layer was discarded and the platelet pellet was gently resuspended in 1 mL of modified HEPES/Tyrode buffer (129 mM NaCl, 0.34 mM Na_2_HPO_4_, 2.9 mM KCl, 12 mM NaHCO_3_, 20 mM HEPES, 1 mM MgCl_2_, 5 mM glucose pH 7.3) containing 150 μL of ACD. An additional 10 mL of modified HEPES/Tyrode buffer containing ACD was added, and the platelets were washed once and then separated by centrifugation at 1,000 × g for 15 min at room temperature. The platelet pellet was resuspended in modified HEPES/Tyrode buffer and adjusted to a concentration of 5 × 10^5^ platelets/mL.

### Neutrophils isolation

Whole blood (collected in 3.2% sodium citrate) from healthy donors was diluted in an equal volume of PBS, and 10 mL were layered over 5 mL of HISTOPAQUE solution (10771, Sigma Aldrich), and centrifuged at 400 × g for 40 min at room temperature. The lower interphase, which contained the granulocytes, was collected and transferred to a 15-mL Falcon tube and resuspended in 10 mL of ammonium chloride lysis buffer (1.7 M NH_4_Cl, 0.1 M KCO_3_, 9.9 × 10^−4^ M EDTA) to lyse red blood cells. Lysis was carried out twice followed by centrifugation for 10 min at 400 × g. Neutrophils were washed with PBS and resuspended at 1 x 10^6^ cells/mL in high glucose DMEM (GIBCO). Neutrophils were kept on ice.

### *In vitro* NET formation

Neutrophils (5 × 10^4^) were treated with 5 nM PMA, platelets (5 × 10^5^), or platelets activated with 1.3 μg/mL collagen. In selected experiments, the neutrophils were pretreated with dipetalodipin (1 μM) or triplatin (1 μM) prior to stimulation. Cells were seeded onto 13 mm cover-slips (Glasscyto) and incubated for 2 h, at 37°C (except for PMA-treated cells which were incubated for 3 h) in DMEM. Cells were fixed with 500 μL of 4% PFA for 10 min, washed 3 times with PBS and incubated for 10 min with blocking solution (PBS, 10% FBS, 5 mg/ml BSA). After blocking, the samples were incubated with goat polyclonal anti-human histone H3 antibody at a 1:50 dilution in blocking solution. Samples were washed 3 times with blocking solution, incubated for 2 h with rabbit anti-goat IgG labeled with Alexa 488 at a 1:500 dilution and Hoechst 33342 at a 1:1000 dilution for NETs visualization, and analyzed under a confocal microscope (Leica, Confocal Microscope LEICA DMI4000 TCS SPE, 20x). Images analyses were performed using the Image J software (NIH).

### Animals

Balb/c mice (both sexes) were housed under controlled temperature (24 ± 1°C) and light (12 h light starting at 7:00 a.m.) conditions, and all experiments were conducted in accordance with the standards of animal care defined by the Institutional Committee (Institute of Medical Biochemistry, Federal University of Rio de Janeiro).

### FeCl_3_-induced carotid artery thrombosis in mice

Balb/c mice were anesthetized with intramuscular xylazin (16 mg/kg) followed by ketamine (100 mg/kg). The right common carotid artery was isolated via a midline cervical incision, and the blood flow was monitored continuously using a 0.5VB doppler flow probe coupled to a TS420 flowmeter (Transonic Systems, Ithaca, NY, USA) as described previously [20]. Thrombus formation was induced by applying a piece of filter paper (1 × 2 mm) saturated with 7.5% FeCl_3_ solution on the adventitial surface of the artery for 3 min. Mean carotid artery blood flow was monitored for 60 min or until stable occlusion occurred (defined as a blood flow of 0 ml/min for ≥ 5 min), at which time the experiment was terminated. Dipetalodipin or triplatin (0.2 or 0.5 mg/kg) or phosphate-buffered saline (PBS) was injected in the vena cava 15 min before injury.

### Collagen/epinephrine-induced pulmonary thromboembolism

Mice were anesthetized as described above. Dipetalodipin or triplatin (0.5 or 2.0 mg/kg) or PBS was slowly injected into the inferior vena cava 15 min prior to the challenge. A mixture of 0.8 mg/kg collagen and 60 μg/kg epinephrine was then injected into the inferior vena cava. Animals that remained alive after 30 min were considered to be survivors.

### Tail bleeding assay

Mice were anesthetized as described above and injected intravenously with dipetalodipin, triplatin (0.5 or 2.0 mg/kg) or PBS in 100 μL volumes. After 15 min, the distal 2 mm segment of the tail was removed and immediately immersed in 40 mL distilled water warmed to 37°C. The samples were properly homogenized and the absorbance was determined at 540 nm to estimate the hemoglobin content. No animal was allowed to bleed for more than 30 min.

### Statistical analysis

All of the statistical analyses were performed using GraphPad Prism 5 (GraphPad Software). One-way analysis of variance (ANOVA) complemented by Tukey's post hoc test was used for comparisons between the test groups. The arterial thrombosis experiments were analyzed by one-way ANOVA with the post hoc Dunnett. The log-rank test was used for the comparison of survival curves. Differences were considered significant when P<0.05. The results were expressed as the mean ± standard error.

## Results

### Dipetalodipin and triplatin abolish the collagen-mediated acceleration of PRP clotting

**Fig 1:**
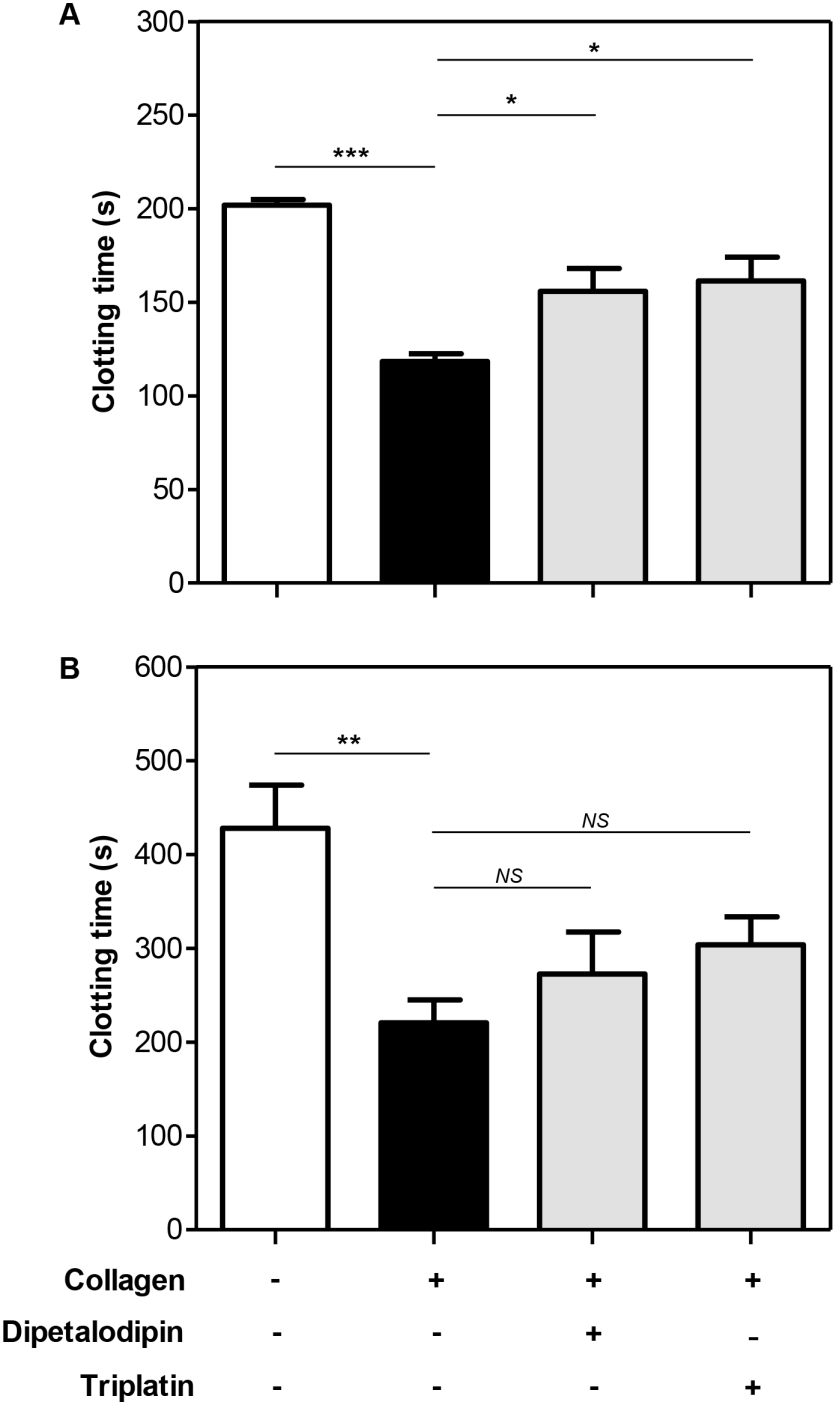
Dipetalodipin and triplatin abolish the collagen-mediated acceleration of PRP clotting. Human citrated-anticoagulated (A) PRP or (B) PPP supplemented with PC/PS was pretreated with dipetalodipin (1 μM) or triplatin (2 μM). Preparations were then incubated with collagen (50 μg/ml, final concentration) or vehicle solvent (control) and activated with 16.6 mM CaCl_2_. Mean ± SEM (n = 5); *P < .05; **P < .01; ***P < .001; NS, non-significant; analysis of variance (ANOVA) with Tukey's posttest.

Because dipetalodipin and triplatin inhibit platelet aggregation induced by low concentrations of collagen, we first evaluated their effect in counteracting the collagen-mediated acceleration of human plasma clotting. Coagulation experiments were performed using either platelet rich plasma (PRP) or platelet poor plasma (PPP). Consistent with previous reports collagen accelerated plasma clotting [19,21], regardless of whether platelets or phospholipids were present in the procoagulant lipid surface (Fig 1A and 1B). However, dipatelodipin and triplatin only abolished this effect in the presence of platelets (Fig 1A). Thus, as shown in Fig 1A, dipetalodipin (1 μM) or triplatin (2 μM) prolonged PRP clotting by 1.3-fold compared to collagen measurements (155.9 ± 12.2 s and 161.4 ± 12.8 s versus 118.5 ± 4.0 s). Addition of collagen to PPP also resulted in a significant shortening of the clotting time, but this effect was not abolished by either dipetalodipin or triplatin (Fig 1B).

### Dipetalodipin and triplatin inhibit the formation of NETs *in vitro*

**Fig 2:**
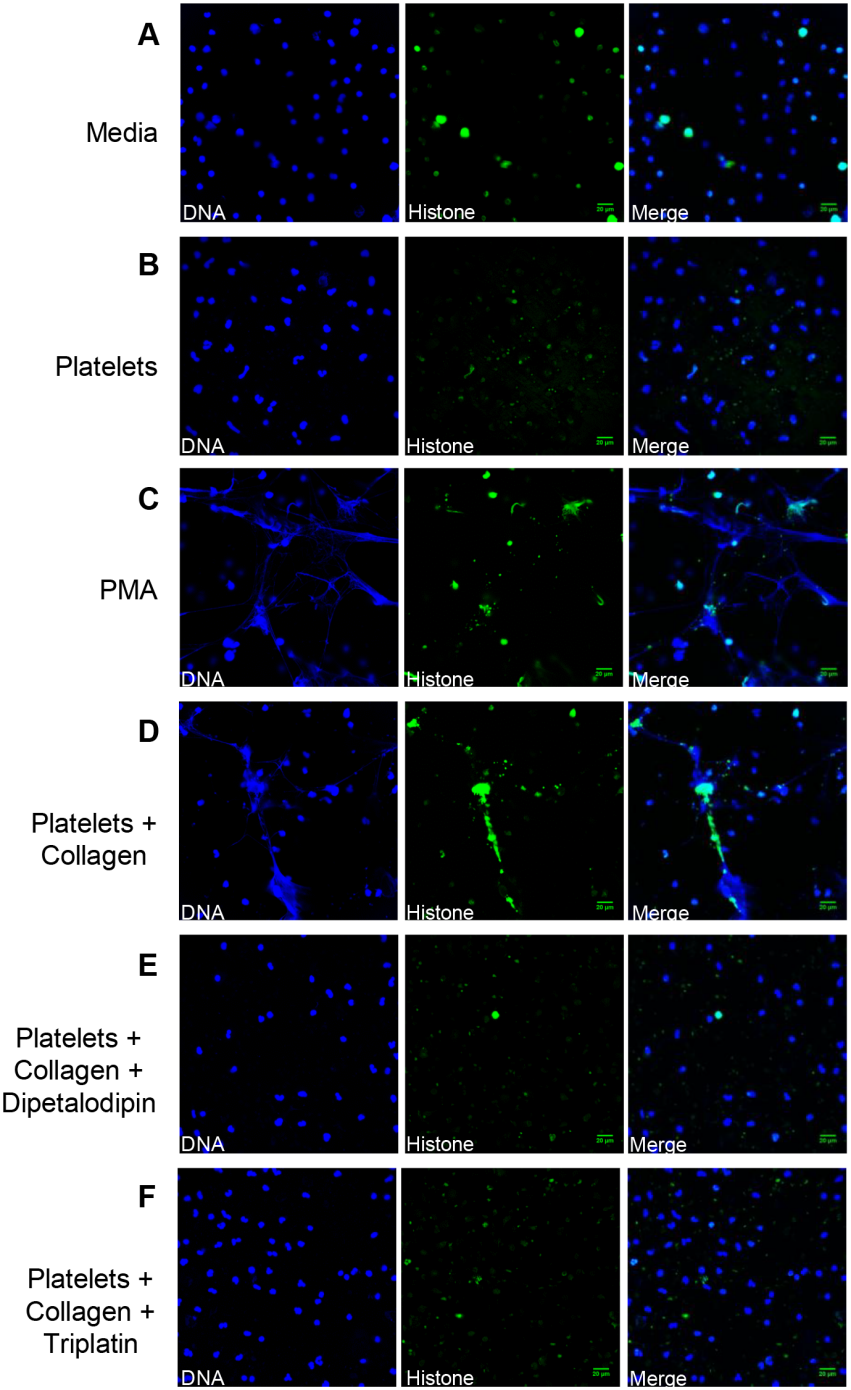
Dipetalodipin and triplatin inhibit platelet-mediated NET formation. (A-F) Representative images from the immunofluorescence staining for neutrophil activation. NET formation was visualized via confocal microscopy using antibodies against DNA (blue) and citrullinated histones (green), as described in the Materials and methods section. No NET formation was apparent for treatment with (A) culture medium or (B) resting platelets. (C) PMA (5 nM) was used as positive control for the formation of NETs. (D) Treatment with platelets previously activated by collagen (1.3 μg/mL) elicited the formation of NETs. Neutrophil incubation with platelets previously activated by collagen in the presence of (E) dipetalodipin (1 μM) or (F) triplatin (1 μM) did not elicit the formation of NETs. Scale bar: 20 μm.

It has been shown that TXA2 produced by activated platelets is a potential mediator in NET formation [18]. To examine the effects of dipetalodipin and triplatin on this response, neutrophils were exposed to platelets that were previously treated with collagen in the presence or in the absence of dipetalodipin or triplatin. NETs were further identified by the co-localization of extracellular DNA and citrullinated histones. Unstimulated neutrophils (Fig 2A) as well as neutrophils treated with resting platelets (Fig 2B) or collagen alone (S1 Fig) showed negligible release of NETs. In contrast, treatment of neutrophils with PMA, a positive control for NET formation, induced a strong response (Fig 2C). Exposure of the neutrophils to platelets that were previously activated with collagen also evoked robust NET formation (Fig 2D), an event that was dramatically inhibited by either dipetalodipin or triplatin (1 μM, Fig 2E and 2F, respectively).

### Dipetalodipin and triplatin display effective antithrombotic activity *in vivo*

**Fig 3:**
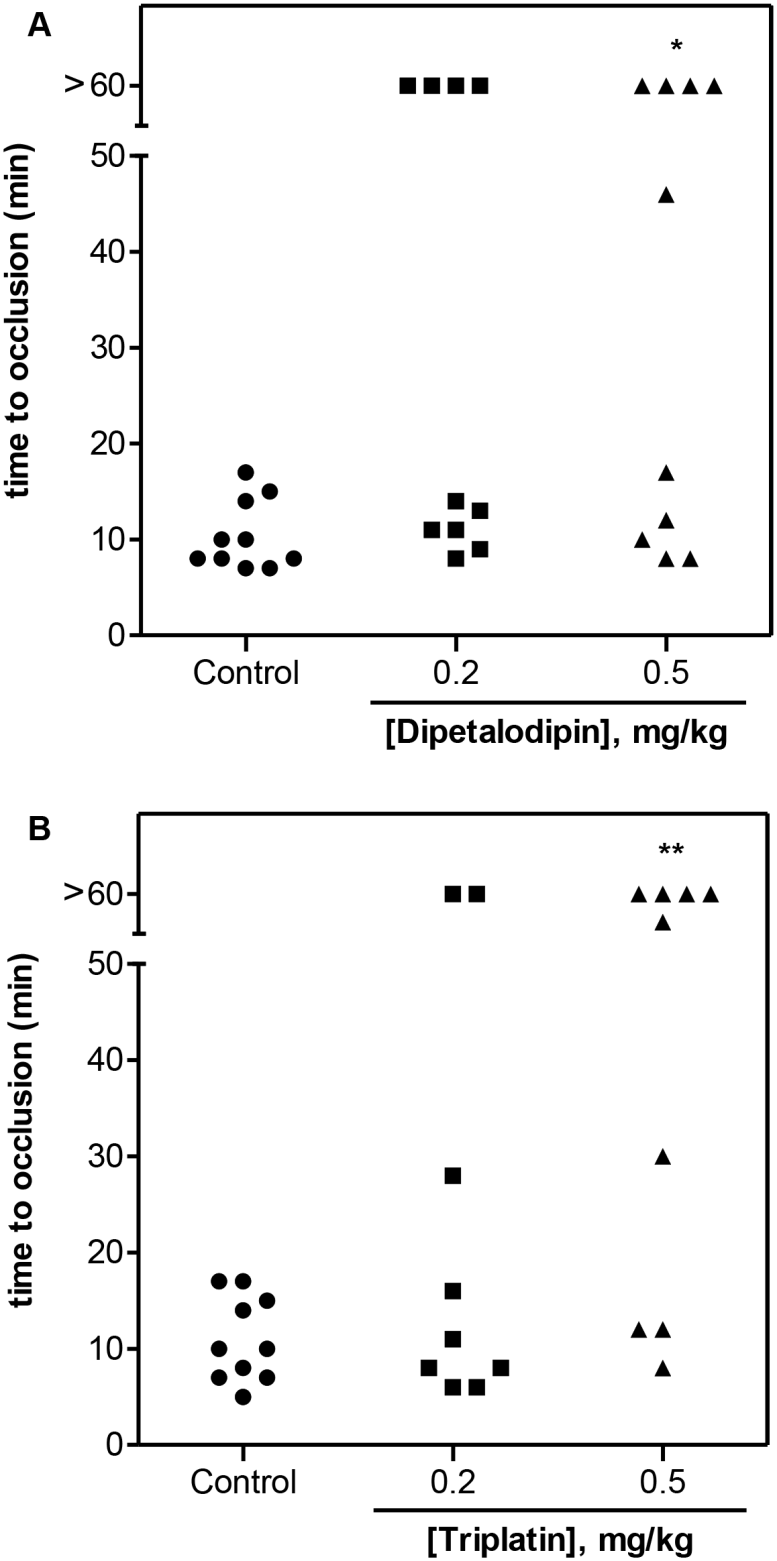
Dipetalodipin and triplatin are antithrombotic *in vivo*. Thrombosis was induced in the carotid artery of mice via local application with 7.5% FeCl_3_. Blood flow was monitored with a perivascular flow probe for 60 min or until stable occlusion occurred. (A) Dipetalodipin or (B) triplatin was injected into the caudal vein 15 min before injury. Each symbol represents one individual. *P < 0.05 vs control, **P < 0.01 vs control; ANOVA with the Dunnett posttest.

The *in vivo* antithrombotic activity of dipetalodipin and triplatin was evaluated by employing two murine models of experimental thrombosis. First, the effect of the TXA2-binding proteins on thrombus formation was assessed using a FeCl_3_-induced carotid artery injury [20]. Thrombus formation was estimated using a Doppler flow probe that allows for the monitoring of carotid blood flow until the vessel occludes, or for up to 60 min if occlusion does not occur. Fig 3 shows that 7.5% FeCl_3_ applied on top of the carotid artery resulted in a reproducible occlusive thrombosis (all animals showed complete vessel occlusion within 20 minutes). Time to occlusion was not statistically significant between control mice and the mice treated either with 0.2 mg/kg dipetalodipin or triplatin, although 4 out of 10 dipetalodipin-treated mice and 2 out of 9 triplatin-treated mice were resistant to arterial occlusion (Fig 3). It is possible that thrombosis induction at this FeCl_3_ concentration is less sensitive for the subtle effect of low doses of dipetalodipin and triplatin. In contrast, treatment with 0.5 mg/kg of either dipetalodipin or triplatin produced significant resistance to thrombosis, as most animals showed no occlusion over the 60 min period (Fig 3).

**Fig 4:**
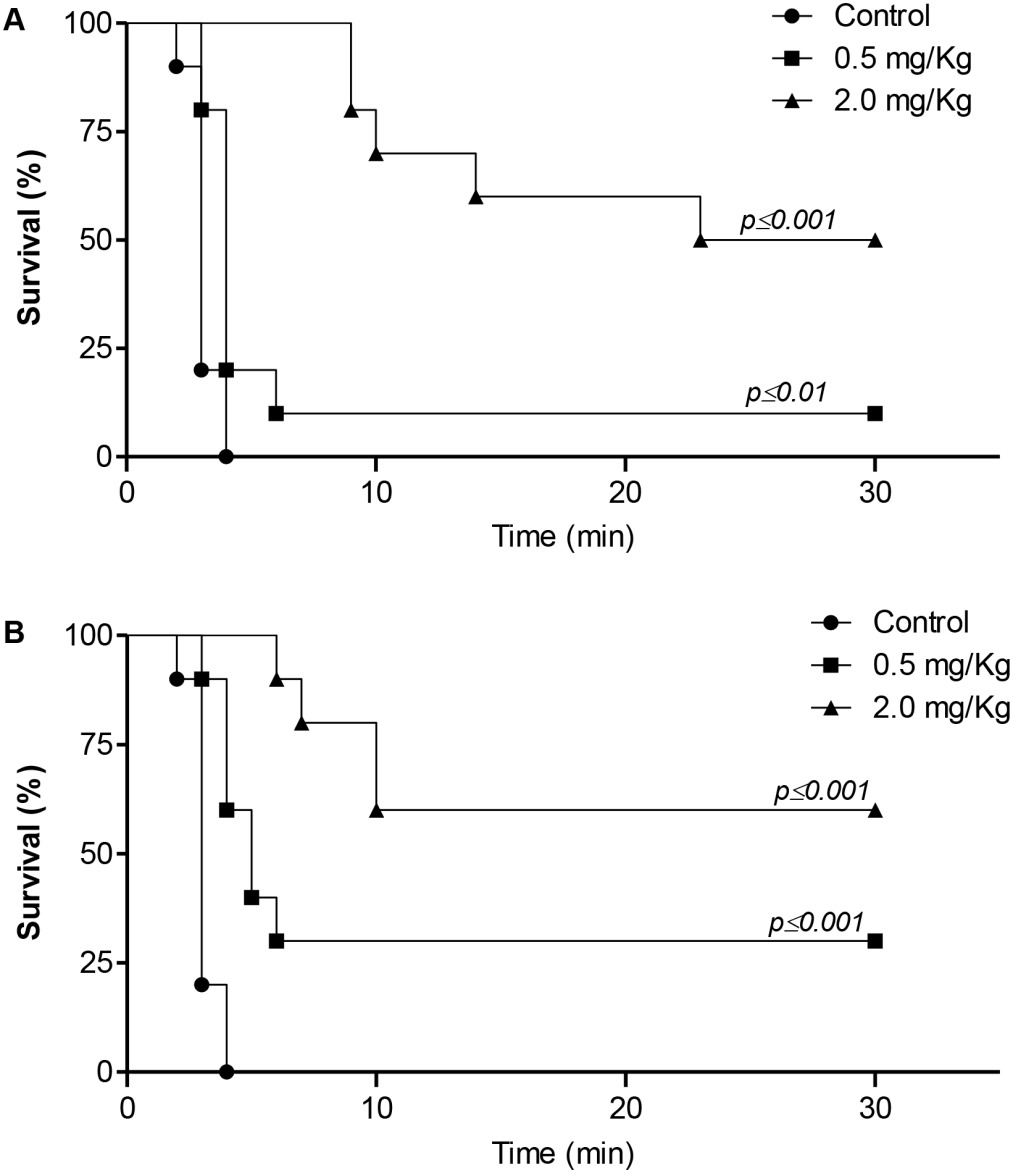
Effect of dipetalodipin and triplatin on the pulmonary embolism model. (A-B) Kaplan-Meier survival curves. Mortality associated with i.v. injection of collagen (0.8 mg/kg) and epinephrine (60 μg/kg) after administration of PBS, (A) dipetalodipin or (B) triplatin. Animals still alive 30 min after the challenge were considered survivors. **P < 0.01 vs control (log-rank test).

The efficacy of dipetalodipin and triplatin in inhibiting thrombus formation was further measured in a murine model of lethal pulmonary thromboembolism, induced by intravenous infusion of collagen and epinephrine. All of the mice treated with vehicle (PBS, 10 out of 10) died within 5 min of collagen/epinephrine infusion (Fig 4). In contrast, the two groups of dipetalodipin—treated mice were significantly protected from death, with up to 50% of the mice surviving the challenge at the highest dose (2.0 mg/kg) (Fig 4A). When triplatin was administered prior to the collagen/epinephrine infusion, we observed a dose-dependent increase in the survival percentage (30% at 0.5 mg/kg and 60% at 2.0 mg/kg triplatin) (Fig 4B). Analysis of the histological sections of the lung tissues confirmed the presence of massive pulmonary thrombosis in PBS-treated mice, compared with the control mice or animals that were treated with either dipetalodipin or triplatin prior to the collagen/epinephrine challenge (S2 Fig).

**Fig 5:**
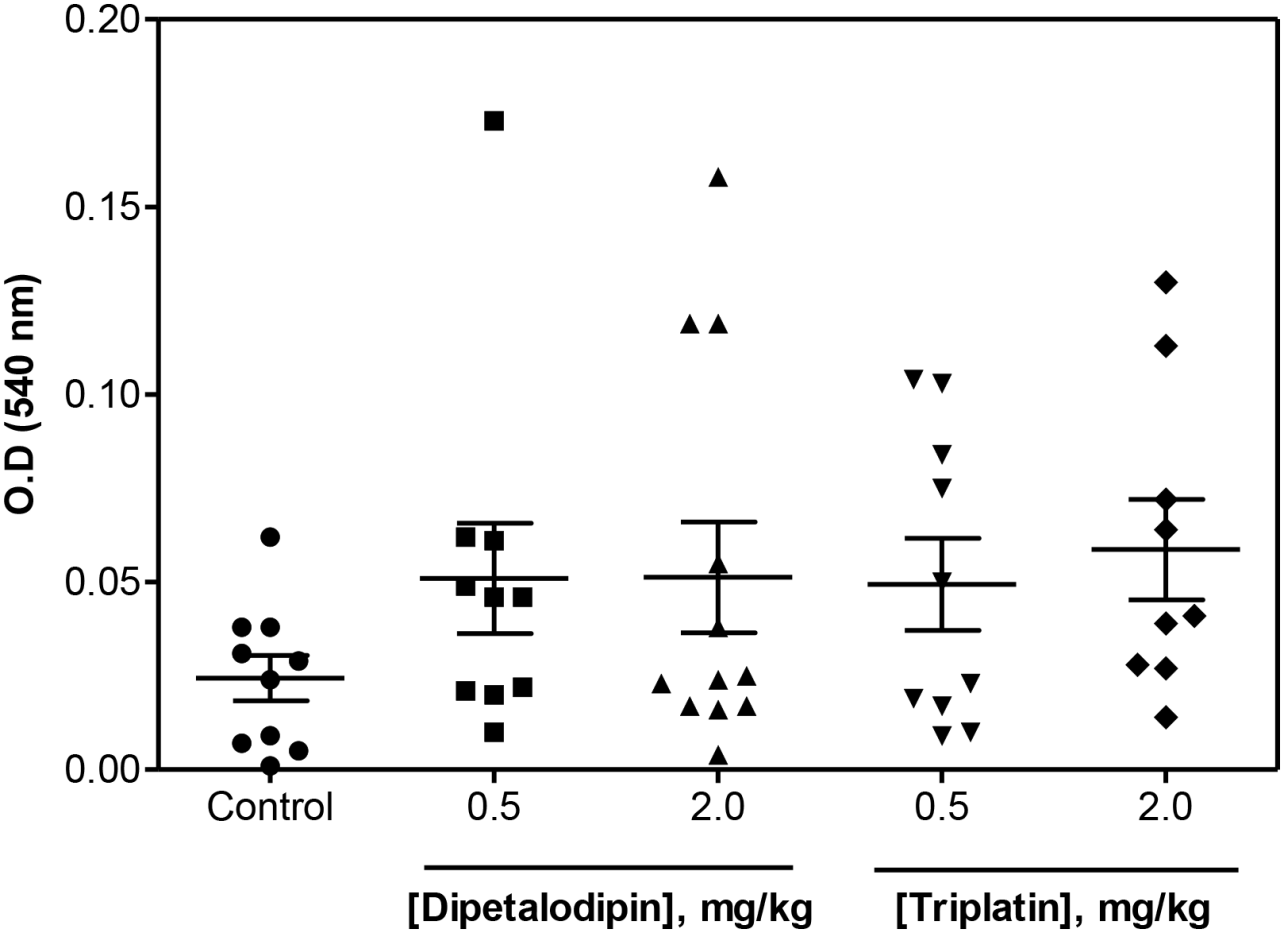
Bleeding effect by the transection model. PBS (control), dipetalodipin or triplatin was administered intravenously and allowed to circulate for 15 min. Blood loss was determined as a function of the hemoglobin concentration in the water (absorbance at 540 nm). Each symbol represents one individual (ANOVA with Tukey's posttest).

The effects of dipetalodipin and triplatin in bleeding were estimated using the tail transection method. Fig 5 shows that bleeding was not significantly increased in the presence of antithrombotic concentrations of either of the inhibitors compared with mice receiving PBS. Dipetalodipin and triplatin did not produce bleeding, even at higher doses (2.0 mg/kg).

## Discussion

The systematic study and characterization of proteins from the saliva of blood-feeding arthropods constitutes a strategy for identifying new exogenous inhibitors of hemostasis [5,22]. Several hematophagous salivary inhibitors of platelet function have been identified, including enzyme inhibitors, NO-releasing molecules, integrin antagonists, apyrases, collagen-binding proteins and molecules that bind biogenic amines [6,8]. Among the platelet inhibitors, members of the lipocalin family have been shown to bind to and remove pro-aggregatory amines such as ADP [23], epinephrine and serotonin [24] and eicosanoids [9,10]. Dipetalodipin and triplatin are proteins that exhibit a unique mechanism of antiplatelet action that consists of a direct interaction with prostanoids, such as TXA2, preventing their biological effect [9,10]. In this report, we demonstrate that dipetalodipin and triplatin prevent platelet-mediated NETs formation *in vitro* and display antithrombotic activity *in vivo*. Both lipocalins are abundantly expressed in the salivary gland, and account for approximately 30% of total salivary lipocalins. Assuming a molecular mass of ∼20 kDa for dipetalodipin and triplatin, and the release of 50% of the salivary contents (∼1 μg/salivary gland pair) upon feeding, a concentration of at least 1 μM of the inhibitor could exist in the feeding environment (∼15 μl); this concentration is clearly in the range required for inhibition of NETs-formation observed *in vitro*.

Upon activation, neutrophils release granule proteins, DNA and histones to form neutrophil extracellular traps (NETs) [25]. NETs formation has been recognized as an important event against pathogens [26]. In addition, *in vitro* and *in vivo* studies provide strong evidence that NETs promote thrombus formation by stimulating platelet aggregation, thrombin generation and contact pathway activation [15,27]. Of note, DNAse treatment inhibits venous thrombosis in mice [28], reinforcing the hypothesis that NETs act as prothrombotic scaffolds for the recruitment of platelets and fibrin deposition during thrombus formation *in vivo* [15,27,28]. Furthermore, it was recently demonstrated that activated platelets induce the formation of NETs [18,28,29]. Our experiments demonstrate that dipetalodipin and triplatin reduce the formation of NETs *in vitro*, indicating that TXA2 produced by activated platelets is required for this process. In this context, inhibition of the TXA2 receptor or pharmacologic inhibition of platelet activation by aspirin impairs NETosis [18]. In addition, platelets contribute to neutrophil activation and NET formation in a murine model of transfusion-related acute lung injury, a process mediated by prostanoids because aspirin has a protective effect [18]. This finding suggests that the antithrombotic effect of dipetalodipin and triplatin may be due to, at least in part, the reduction in platelet-assisted NET formation. Other salivary inhibitors such as agaphelin, also inhibits NETs formation and prevent thrombosis without impairing hemostasis [30]. Interestingly, it has been recently reported that degrading of NETs by *Leishmania infantum* prevents their killing by neutrophils [31]. In this context, saliva components that modulate the inflammatory responses [32,33] as well as those capable to prevent NETs formation may contribute to evasion of parasites, such as trypanosomatids, from host’s innate immune responses.

TXA2, as well as ADP, is secreted by activated platelets and acts as an important second wave mediator for platelet activation and collagen-mediated aggregation. These mediators, which are released by activated platelets at the site of vascular injury are crucial for the establishment and maintenance of the thrombus [2,4]. Lack of TXA2 receptors results in thrombus instability and prolonged bleeding times [34,35]. Likewise, inhibition of TXA2 synthesis by aspirin results in reduced thrombus formation and high rates of embolization *in vivo* [36]. In addition, aspirin or TXA2 receptor antagonists reduce collagen-induced thrombus formation in *in vitro* flow experiments [36]. Accordingly, dipetalodipin and triplatin display antithrombotic activity *in vivo*, as demonstrated using a FeCl_3_-induced carotid artery injury model. These results are consistent with the observation that mice deficient in the TXA2 receptor exhibit prolonged occlusion time in the same thrombosis model used in our study [35]. Inhibition of TXA2 by dipetalodipin and triplatin may also explain their protective effects in the pulmonary thromboembolism assay, which is sensitive to compounds with antiplatelet activities [37], including agents that inhibit the synthesis or action of TXA2 [38,39]. Notably, no significant bleeding was observed at antithrombotic doses of dipetalodipin and triplatin. Remarkably, other salivary gland-derived proteins display a similar effect: nitrophorin 2 and desmolaris, which inhibit the contact pathway of blood coagulation, are effective antithrombotic agents *in vivo* while promoting minor hemorrhagic effects in the tail transection bleeding assay [40,41]. This contrasts with other clotting inhibitors, such as the factor Xa inhibitor lufaxin [42], which target downstream coagulation steps.

A well-established mechanism for TXA2 production is through the binding of vWF to the platelet GPIb-IX-V complex. *In vitro*, this interaction initiates a cellular signaling cascade that elicits TXA2 production, GP-IIbIIIa exposure and platelet aggregation [35,43]. *In vivo*, deletion of components in the signaling cascade initiated by vWF increases the occlusion time of the carotid artery in the FeCl_3_-induced injury [35,44]. Additionally, the platelet-collagen interaction, mediated by GPVI, is involved in TXA2 production [45,46]. Our data demonstrated that dipetalodipin and triplatin counteract the collagen-mediated acceleration of human platelet-rich plasma clotting. Therefore, antithrombotic effect of dipetalodipin and triplatin impairs the downstream responses elicited by the platelet-collagen and platelet-vWF interactions, resulting in impaired availability of TXA2 which reportedly plays an important role in arterial thrombus consolidation.

Altogether, we demonstrated that salivary TXA2-binding proteins, dipetalodipin and triplatin, are capable to prevent platelet-mediated NETs formation *in vitro*. Notably, both molecules inhibited arterial thrombosis without promoting excessive bleeding in mice models. Our results provide new insight into the antihemostatic effects of TXA2-binding proteins and may have important significance in elucidating the mechanisms of saliva to avoid the innate immune system.

## Supporting Information

S1 Fig Negligible NET formation in human neutrophils incubated with collagen. Adherent neutrophils were incubated with collagen (1.3 μg/mL) for 3 h at 37°C. NET formation was visualized via confocal microscopy using antibodies against DNA (blue) and citrullinated histones (green), as described in the Materials and methods section. Neutrophil incubation with collagen did not elicit the formation of NETs. Scale bar: 20 μm.
(PDF)

S2 Fig Microscopic examination of lungs of mice challenged with collagen and epinephrine. Hematoxylin and eosin-stained lung sections of (A) healthy lungs, (B) PBS-treated mice, (C) dipetalodipin-treated mice (2 mg/kg) or (D) triplatin-treated mice (2 mg/kg). Animals were euthanized 5 min after the collagen and epinephrine injection. Representative images from each condition are shown in the figure. Arrows indicate fibrin thrombi. Bars represent 100 μm.
(PDF)
