# Supplementary material for: Salivary Thromboxane A2-Binding Proteins from Triatomine Vectors of Chagas Disease Inhibit Platelet-Mediated Neutrophil Extracellular Traps (NETs) Formation and Arterial Thrombosis
Source: PLoS Negl Trop Dis. 2015 Jun 25;9(6):e0003869. doi: 10.1371/journal.pntd.0003869 (PMC4482233; doi:10.1371/journal.pntd.0003869)
Supplement: S1 Fig — Adherent neutrophils were incubated with collagen (1.3 μg/mL) for 3 h at 37°C. NET formation was visualized via confocal microscopy using antibodies against DNA (blue) and citrullinated histones (green), as described in the Materials and methods section. Neutrophil incubation with collagen did not elicit the formation of NETs. Scale bar: 20 μm. (PDF) [file pntd.0003869.s001.pdf]

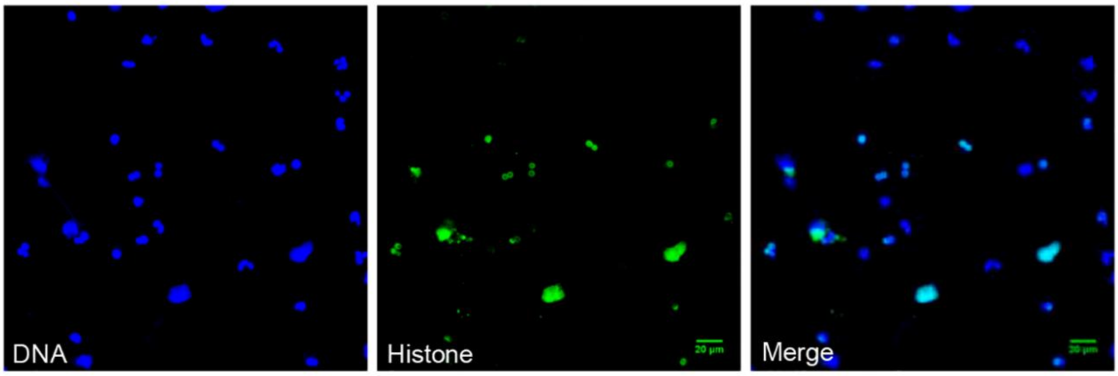

S1 Fig. Negligible NET formation in human neutrophils incubated with collagen. Adherent neutrophils were incubated with collagen (1.3 µg/mL) for 3 h at 37ºC. NET formation was visualized via confocal microscopy using antibodies against DNA (blue) and citrullinated histones (green), as described in the Materials and methods section. Neutrophil incubation with collagen did not elicit the formation of NETs. Scale bar: 20 µm.
