# Supplementary material for: Salivary Thromboxane A2-Binding Proteins from Triatomine Vectors of Chagas Disease Inhibit Platelet-Mediated Neutrophil Extracellular Traps (NETs) Formation and Arterial Thrombosis
Source: PLoS Negl Trop Dis. 2015 Jun 25;9(6):e0003869. doi: 10.1371/journal.pntd.0003869 (PMC4482233; doi:10.1371/journal.pntd.0003869)
Supplement: S2 Fig — Hematoxylin and eosin-stained lung sections of (A) healthy lungs, (B) PBS-treated mice, (C) dipetalodipin-treated mice (2 mg/kg) or (D) triplatin-treated mice (2 mg/kg). Animals were euthanized 5 min after the collagen and epinephrine injection. Representative images from each condition are shown in the figure. Arrows indicate fibrin thrombi. Bars represent 100 μm. (PDF) [file pntd.0003869.s002.pdf]

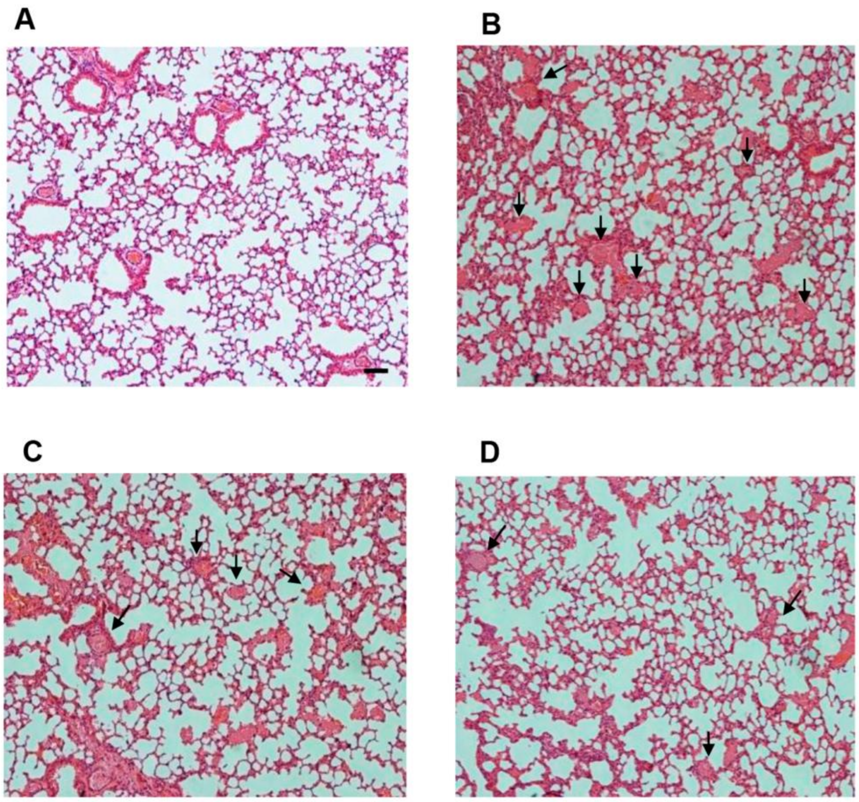

S2 Fig. Microscopic examination of lungs of mice challenged with collagen and epinephrine. Hematoxylin and eosin-stained lung sections of (A) healthy lungs, (B) PBS-treated mice, (C) dipetalodipin-treated mice (2 mg/kg) or (D) triplatin-treated mice (2 mg/kg). Animals were euthanized 5 min after the collagen and epinephrine injection. Representative images from each condition are shown in the figure. Arrows indicate fibrin thrombi. Bars represent 100 μm.
